# Enhancing Osteoconduction of PLLA-Based Nanocomposite Scaffolds for Bone Regeneration Using Different Biomimetic Signals to MSCs

**DOI:** 10.3390/ijms13022439

**Published:** 2012-02-22

**Authors:** Gabriela Ciapetti, Donatella Granchi, Valentina Devescovi, Serena R. Baglio, Elisa Leonardi, Desirèe Martini, Maria Jesus Jurado, Beatriz Olalde, Ilaria Armentano, Josè M. Kenny, Frank X. Walboomers, Josè Inaki Alava, Nicola Baldini

**Affiliations:** 1 Laboratory for Orthopaedic Pathophysiology and Regenerative Medicine, Istituto OrtopedicoRizzoli, Bologna 40136, Italy; E-Mails: donatella.granchi@ior.it (D.G.); valentina.devescovi@gmail.com (V.D.); serena.baglio@ior.it (S.R.B.); elisa.leonardi4@unibo.it (E.L.); nicola.baldini@ior.it (N.B.); 2 Dipartimento di Scienze Anatomiche e dell’Apparato Locomotore, University of Bologna, Bologna 40136, Italy; E-Mail: desiree.martini@unibo.it; 3 Health Unit, INASMET-Tecnalia, San Sebastian E-20009, Spain; E-Mails: mariaje.jurado@tecnalia.com (M.J.J.); beatriz.olalde@tecnalia.com (B.O.); ialava@bculinary.com (J.I.A.); 4 Materials Engineering Centre, UdR INSTM, NIPLAB, University of Perugia, Terni 05100, Italy; E-Mails: ilaria.armentano@lnl.infn.it (I.A.); kenny@unipg.it (J.M.K.); 5 Department of Biomaterials, Radboud University, Nijmegen Medical Centre, Nijmegen 6525 GA, The Netherlands; E-Mail: f.walboomers@dent.umcn.nl

**Keywords:** bone tissue engineering, biomimetic nanocomposites, mesenchymal stem cell

## Abstract

In bone engineering, the adhesion, proliferation and differentiation of mesenchymal stromal cells rely on signaling from chemico-physical structure of the substrate, therefore prompting the design of mimetic “extracellular matrix”-like scaffolds. In this study, three-dimensional porous poly-L-lactic acid (PLLA)-based scaffolds have been mixed with different components, including single walled carbon nanotubes (CNT), micro-hydroxyapatite particles (HA), and BMP2, and treated with plasma (PT), to obtain four different nanocomposites: PLLA + CNT, PLLA + CNTHA, PLLA + CNT + HA + BMP2 and PLLA + CNT + HA + PT. Adult bone marrow mesenchymal stromal cells (MSCs) were derived from the femur of orthopaedic patients, seeded on the scaffolds and cultured under osteogenic induction up to differentiation and mineralization. The release of specific metabolites and temporal gene expression profiles of marrow-derived osteoprogenitors were analyzed at definite time points, relevant to *in vitro* culture as well as *in vivo* differentiation. As a result, the role of the different biomimetic components added to the PLLA matrix was deciphered, with BMP2-added scaffolds showing the highest biomimetic activity on cells differentiating to mature osteoblasts. The modification of a polymeric scaffold with reinforcing components which also work as biomimetic cues for cells can effectively direct osteoprogenitor cells differentiation, so as to shorten the time required for mineralization.

## 1. Introduction

The goal of tissue engineering is the regeneration of an adult damaged tissue, *i.e.*, the restoration of the physical and mechanical nature of the native tissue [1,2]. Using an “extracellular matrix” (ECM)-like resorbable scaffold that cells can attach to, proliferate into, and migrate across, the damaged tissue may be replaced by the construct, which in turn is slowly resorbed and substituted by new functional tissue [3]. Therefore the process of tissue engineering starts with the sourcing of the relevant cells, to end with the full integration of the functional regenerated tissue into the host.

Inflammation and scarcity of pluripotent stem cells or progenitors at the injury site could hamper the proper bone healing mechanisms; therefore the engraftment of mesenchymal stem cells has been shown to provide a potent cell therapy for tissue repair. Though the cells potentially useful include those derived from autologous, allogeneic or, possibly, xenogeneic sources, autologous cells are preferred in terms of availability and safety [4]. The degree of cell manipulation *ex vivo* will depend on the origin of the cells and the complexity of the tissue, but due to the artificial conditions of an *in vitro* culture, the phenotype of the cells has to be controlled during cell expansion [5].

To be engrafted in the living tissue adherent cells require a supporting structure, either a scaffold, a matrix, or a membrane, where new tissue will be generated in response to molecular and mechanical signals. The cell-material system is referred to as a construct, which is generated *ex vivo*, and has to be fully accepted by the host to achieve an effective regeneration of functional tissue [6].

Bone marrow-derived mesenchymal stromal cells (MSCs) are able to differentiate *ex vivo* along mesenchymal lineages, following *in vitro* expansion under the influence of specific chemicals [7]. But differentiation of MSCs is also governed by other stimuli, such as chemistry, micro- and nano-topography, and rigidity of the substrate, as they usually do *in vivo* when embedded in the ECM. Therefore, an artificial scaffold for bone should be designed to be instructive to MSCs to undergo osteogenic maturation whereas accomplishing its mechanical tasks during the entire period of bone repair [8]. As a consequence, a biodegradable biomimetic material that induces or promotes significant new bone formation by osteogenic cells at an injured site is the desirable engineered substrate for orthopaedics [9,10].

Since natural bone is a reinforced organic/inorganic composite, *i.e.*, collagen/hydroxyapatite (HA) composite, a polymer-based HA composite is a promising material for use as bone implant. Among biodegradable polymers used for bone engineering, poly-lactic acid (PLLA) has great modeling properties, controlled degradation with time and good tissue compatibility [11]. Nevertheless, the mechanical and surface properties of this material are unable to bear heavy loads and to stimulate cell adhesion, respectively. The addition of micro- and nanoparticles or fibers to the polymeric matrix may improve mechanical properties, and surface functionalization may promote bone cell adhesion [12].

In this study, a micro-macroporous PLLA matrix was synthesized, and then functionalized with different biomimetic signals, including (i) dispersion of single-wall carbon nanotubes (SWCNT, CNT for simplicity) and micro-hydroxyapatite particles (HA) in the matrix, to mimic bone constituents; (ii) plasma deposition to change the surface hydrophobicity; and (iii) loading of bone morphogenetic protein 2 (BMP2) as an osteogenic inducer.

CNT and HA are supposed to closely approximate the function of HA crystals and collagen fibers in bone; moreover the release of Ca^2+^ ions from HA should act as a buffer for acidic products of polymer degradation. HA particles have been shown to act as a reinforcing agent, but also as a source of calcium and phosphate easily recognized by osteoblasts as a “natural” mineral phase [13,14].

Plasma-based strategies for selective surface modification have been largely applied to hydrophobic polymeric scaffolds, such as PLLA, and enhanced interaction with cells has been observed [15,16].

Some of the scaffolds under assay have been loaded with BMP2, to serve as delivery vehicles for the growth factor. The bone-promoting activity of BMP2 *in vitro* and *in vivo* has been recognized and approved some years ago [17], even if the biologic potency of such growth factor in enhancing bone formation is still debated [18,19].

Theoretically, each of these signals may activate osteoprogenitor cells and genes, and consequently promote tissue growth and organization at the site of injury [20]. In this study the response of human MSCs to 3D PLLA-based scaffolds with controlled macro- and micro-structures prepared by Thermally Induced Phase Separation technique was assessed. The bioactive behavior was conferred to the polymer matrix by using four different strategies, including CNT and/or HA particles addition, BMP2 loading, and plasma treatment of the surface. The osteoconductive properties of the different scaffolds have been verified by analyzing morphology, biochemistry and gene expression of human MSCs seeded on the scaffolds and cultured under osteogenic induction.

## 2. Results and Discussion

The reduction of time for fracture healing or the treatment of non-unions and joint diseases are current problems in orthopaedics, and regenerative strategies have been shown to have a high potential in favoring bone repair [21–23].

To avoid cell dispersion away from the injured site, MSCs are better delivered *in vivo* using a carrier, such as ceramic granules, fibrin, *etc*., which may also positively interact with the cells [24,25], or a scaffold, which provides a suitable ECM-like environment with signals for cell survival and functions, and may deliver additional growth factors. Several prefabricated polymer-cell constructs have been shown to assist host cells for attachment and subsequent proliferation and differentiation; since cells interact with ECM at both micro- and nano-scale level, the addition of micro- and nano-sized components to bulk materials during matrix manufacturing is likely to enhance cell response [26–28]. In this study the osteoconductive properties of some composites were evaluated by using a combination of morphological, biochemical and molecular assays [4]. Four types of scaffolds were prepared and assayed, including: PLLA + CNT, PLLA + CNT + HA, PLLA + CNT + HA + BMP2, and +PLLA + CNT + HA+ “plasma enhanced chemical vapor deposition (PECVD)-treatment” (PT).

**Figure 1:**
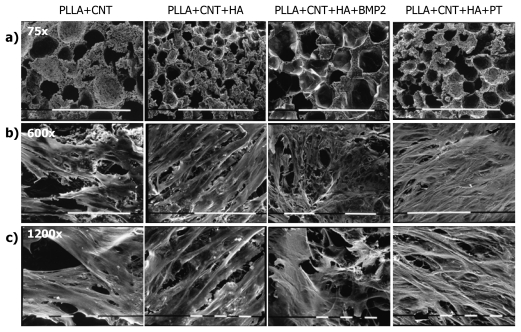
Scanning electron microscopy (SEM) of the porous poly-L-lactic acid (PLLA) scaffolds kept for 7 days in mineralization medium without (**a**, bar = 1 mm) or with mesenchymal stromal cells (MSCs) (**b**, bar = 100 μm; **c**, bar = 10 μm). Images of lane **a** show that the micro/macro-porosity of the composites is slightly different. In lanes **b** and **c** MSCs are seen to spread, proliferate and show intercellular connections on all the composites. On PLLA + carbon nanotube (CNT) the cells do not form a continuous layer. Instead, on PLLA + CNT + micro-hydroxyapatite particles (HA) calcium phosphate nodules are clearly seen, and on PLLA + CNT + HA + plasma (PT) cells are multilayered.

The PLLA-based scaffold prepared in this study had a mean pore diameter of 103 μm, and a mean porosity of 87%, as measured by mercury intrusion porosimetry. Though slight differences in pore distribution, the scaffold architecture was similar (Figure 1, lane a). The Young modulus was 12.5 ± 2.0 MPa and the maximum stress 0.39 ± 0.02 MPa. The hydrophobicity of the scaffolds was a potential drawback for cell inoculation, therefore the scaffolds were pre-wet with serum-added culture medium for two hours.

### 2.1. Cell Culture Characterization

**Figure 2:**
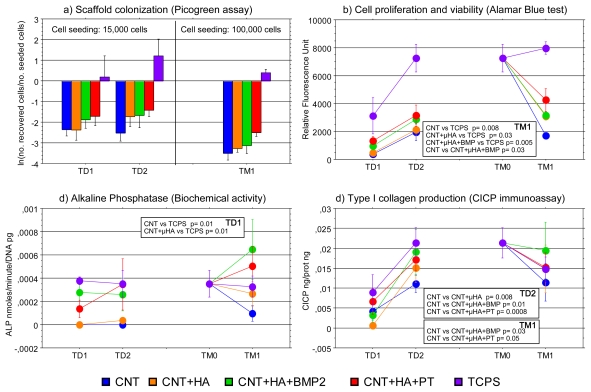
(**a**) Data on the scaffold colonization are expressed as mean of the ratios (“ln” transformed) between number of cells recovered from each scaffold at the different end points and number of seeded cells; (**b**) In agreement with the number of recovered cells, the fluorescence emitted by the Alamar Blue dye is higher in Tissue Culture Plastic Surface (TCPS) cultures. All the cultures show an increase in fluorescence from TD1 to TD2, while from TM0 to TM1 the lowest signal is found in PLLA + CNT samples; (**c**) At TD1, alkaline phosphatase activity is lower in scaffolds than in TCPS, but it increases from TM0 to TM1 so that the enzyme activity at final end point is similar in 2D and 3D cultures; (**d**) The release of type I collagen is higher in 2D cultures, but the addition of bone morphogenetic protein 2 (BMP2) favors the collagen production during the mineralization phase.

The following time points were considered for cell culture characterization: (i) in differentiation medium: 7 days (TD1) and 14 days (TD2) after seeding on nanocomposites; (ii) in mineralization medium: before cell seeding onto nanocomposites (TM0) and after 7 days (TM1). Scanning electron microscopy (SEM) was used to observe morphology and spreading of cells on the scaffolds (Figure 1, b and c); MSCs on “Tissue Culture Plastic Surface” (TCPS) were arranged as monolayers on 2D plastic surface (images not shown), and exhibited an exponential growth rate from TD1 to TD2, because the cells were seeded at low density (14,727 ± 214 cells/scaffold). On the contrary, from TM0 to TM1 a modest increase in cell proliferation was observed, as cells were seeded at higher density (95,333 ± 2,353 cells/scaffold). The cells retrieved from 3D materials at different time points were even lesser in comparison with the number of seeded cells, but the addition of HA to the composite apparently enhanced the number of cells on the scaffolds, which was maximally increased in samples treated with PECVD (Figure 2a).

The differences in cell attachment/proliferation were further confirmed by the Alamar Blue test (Figure 2b). Even if the fluorescent signals emitted by cells were steadily higher when MSCs were grown on TCPS than onto the nanocomposites, the fluorescence emission increased from TD1 to TD2 in all the cultures, which means that cells were proliferating over all the scaffolds. At TM1 significant differences were found between TCPS and all the scaffolds, except for PLLA + CNT + HA + PT. MSCs cultured on PLLA + CNT samples exhibited the lowest signal.

Alkaline phosphatase (ALP) activity was characterized by a large variability among individuals (Figure 2c). At TD1, it was significantly lower in both PLLA + CNT and PLLA + CNT + HA than in TCPS cultures. The enzyme activity of MSCs cultured on TCPS tended to be constant from TD1 to TM1, whilst it increased in cells cultured into the scaffolds after the addition of the mineralization medium, with a peak for PLLA + CNT + HA + BMP2 nanocomposites.

Type I collagen production was high when cells were cultured on TCPS (Figure 2d). Unpredictably, the CICP release in 2D cultures (TCPS) was more variable than in 3D ones; therefore no significant difference was detected when TCPS was considered for statistical comparison. The collagen release tended to increase from TD1 to TD2, and significant differences were recorded when HA was inside the scaffolds. At M1, the addition of HA alone was not enough to support the collagen production, while significant differences in comparison to PLLA + CNT were detected for samples treated with PEVCD or added with BMP2.

Though their *in vivo* safety is still controversial [29], CNT have been largely exploited as reinforcing agents [30], drug delivery [31], and bone repair [32], due to their unique chemico/physical and mechanical properties. In our hands, the CNT-added PLLA had a low osteoconduction ability, since adhesion, proliferation, differentiation and mineralization of MSCs were hampered in comparison to TCPS and the other composites. Such results are in agreement with a recent paper, where purified and dispersed CNT can induce actin bundling and proliferation decrease in cells [33]. The addition of HA micro-particles to the CNT-PLLA composite was a positive signal for MSC adhesion and proliferation. Indeed, the biomimetic role of HA as matrix filler in bone repair has been largely demonstrated *in vitro* and *in vivo* [34,35]. Though not shown in this study, a role played by the increased stiffness of HA-added PLLA *vs.* plain PLLA in inducing spreading and “mesenchymal” migration of MSCs, may be hypothesized [36].

There were several limitations to a comprehensive characterization of MSCs inside the 3D structure, because HA microparticles were interfering with the analysis of the mineral deposited. However, biochemical tests showed that the addition of BMP2 to PLLA + CNT + HA composite promoted alkaline phosphatase (ALP) activity and type I collagen production, and this may be considered a proof of the osteogenic differentiation of MSCs. Concerning the surface treatment, it is well known that changes in matrix hydrophobicity can dramatically alter cell-matrix interactions and in turn have a profound impact on various cellular behaviors such as adhesion, shape, motility, cytoskeletal organization, and differentiation [10]. The plasma-treatment of PLLA scaffolds with oxygen using “plasma enhanced chemical vapor deposition” (PECVD) had a strong impact on the adhesion of osteoprogenitors, thus speeding the formation of a construct suitable for initiating a regenerative process. Plasma treatment of hydrophobic polymers is one of the approaches used for providing micro- and nano-metric alterations of the surface architecture to improve protein and cell adhesion [37,38], and this mechanism is likely to be implied in our samples treated with plasma. Finally, the loading of BMP2 in the PLLA matrix positively influences the late events in osteoblast maturation, *i.e.*, differentiation/mineralization of MSCs. The induction of osteogenic markers by BMP2 administration is recurrently demonstrated in murine progenitor cells, whereas this effect is not reproducibly induced *in vitro* or *in vivo* in bone marrow-derived human MSCs [39–41]. Accordingly to other studies, we found a positive effect on *in vitro* osteogenesis using BMP2 within a “complex” 3D scaffold [42].

### 2.2. Gene Expression Analysis

**Figure 3:**
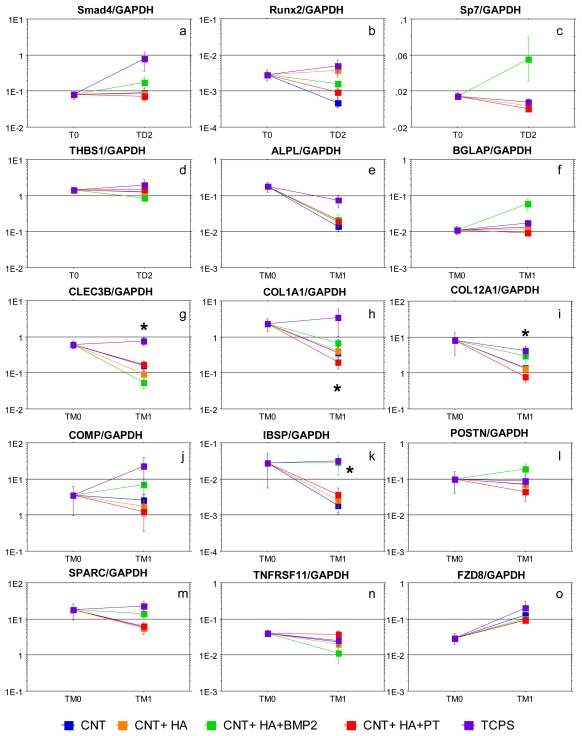
Gene expression analysis. Graphs show the results of the Real Time PCR expressed as mean ± SEM of the ratios among “genes of interest” and GAPDH. The gene expression has been evaluated at T0 (before the seeding on nanocomposites) and TD2, or at TM0 and TM1. The asterisks mean the presence of statistical significant differences at that time point, and p values are referred in the text.

Gene expression analysis was used in order to explore all the cell functions that cannot be easily analyzed using morphological, cytochemical, and biochemical assays. In a previous study we used the microarray technology to define the gene expression patterns underlying the consecutive steps of human adult MSCs differentiating into mature osteoblasts [43]. Whilst more than two-hundred genes with a biological function relevant to osteogenesis were selected, in the current study we focused on (i) some bone-related genes, to analyze the osteoinductive properties of nanostructured scaffolds; (ii) some TGFβ signaling related genes, to highlight the BMP2 activity, a molecule of the TGFβ superfamily of proteins; and (iii) one gene belonging to the Wnt signaling related genes, which is strongly and uniquely upregulated during the mineralization phase (Table 1). The quantitative results of gene expression analysis are shown, taking into account the biological function of the gene of interest, in Figure 3.

Genes belonging to the TGFβ signaling were tested only during the differentiation steps because they are expected to be up-regulated quite early along MSC culture (Figure 3a–d). Generally, the expression of these genes was decreased in cells cultured into the scaffolds. Smad4 increased in MSCs cultured onto TCPS and BMP2-added composites, while no changes were observed in cells colonizing other materials. Runx2 expression was similar in TCPS and PLLA + CNT + HA, while with the other scaffolds it decreased. Sp7 expression was widely influenced by the presence of BMP2, even if the responsiveness varied among individuals. The expression of THBS1, a negative regulator of TGFβ signaling, was not influenced by the culture on composites, even if a lower amount of transcripts was observed in samples with BMP2. Collectively, genes belonging to TGFβ signaling were downregulated when cells were cultured into the scaffolds. The addition of BMP2 limited the decrease in Smad4 expression and favored the expression of SP7, a transcription factor which acts downstream of Runx2 to induce osteoblast differentiation in osteochondroprogenitor cells [44,45]. Although Smads are critical mediators in the TGFβ signaling pathway, BMP2 can activate Smad-independent pathways, including mitogen-activated protein kinases that have distinct roles in regulating alkaline phosphatase and osteocalcin expression in osteoblastic cells [46]. Moreover, a lower amount of THS1 transcripts is observed in samples exposed to BMP2: since it is an inhibitor of bone mineralization and matrix production, its low expression could be considered as a signal favoring osteogenesis [47].

In the first 14 days of culture, the ALPL expression was similar in MSCs on TCPS and MSCs on nanocomposites (data not shown). During the mineralization phase the amount of ALPL transcripts diminished in cells cultured onto the scaffolds, and this decrease was less pronounced in materials with BMP2 (Figure 3e).

During the mineralization period the expression of noncollagenous proteins was lower in MSCs cultured on nanocomposites, with significant differences in IBSP expression found between TCPS and PLLA + CNT (*p* = 0.05), and between TCPS and PLLA + CNT + HA (*p* = 0.05) (Figure 3k). The expression of COMP, SPARC and POSTN did not change significantly in comparison to TCPS (Figure 3j–m, respectively). Generally, the expression of noncollagenous proteins in PLLA + CNT + HA + BMP2 composite was close to that observed in cells cultured on TCPS, and even higher in the case of BGLAP (Figure 3f) and POSTN (Figure 3l). The only exception was CLEC3B, which was significantly less expressed in MSCs on the BMP2-added nanocomposite than in PLLA + HA (*p* = 0.003) (Figure 3g). The expression of COL1A1 and COL12A1 (Figure 3h,i, respectively) was decreased in MSCs on nanocomposites through the mineralization. The negative effect was weakened by the presence of BMP2. At TM1, the collagen expression in PLLA + CNT + HA + BMP2 composites was significantly higher than in PLLA + CNT + HA + PT (COL1A1 *p* = 0.02; COL12A1 *p* = 0.05). Summarizing, the most significant results were obtained by analyzing the expression of bone-related genes, especially during the mineralization phase. A decrease of ALP transcripts was evident in all the scaffolds, but it was less pronounced using PLLA added with BMP2. A correlation between gene expression and biochemical activity was not found, likely due to the high variability of the results.

It is known that bone formation by osteoblastic cells requires the deposition of an extracellular matrix, consisting of collagen, a variety of fibrous and non-fibrous proteins, and glycosaminoglycans, to undergo mineralization upon formation of hydroxyapatite crystals. Type I collagen fibers are involved in aligning the mineral crystals, thus playing a pivotal role in mediating the apatite formation in normally mineralizing vertebrate tissues [48]. Noncollagenous proteins, including BGLAP/osteocalcin [49], CLEC3B/tetranectin [50], IBSP/bone sialoprotein [51], SPARC/osteonectin [52] and POSTN/periostin [53], comprise 10% of the organic matrix of bone. These proteins are expressed in areas of active remodeling, bind the hydroxyapatite crystals, and promote relevant functions in the regulation of mineralization. COMP/Cartilage oligomeric matrix protein is a prominent noncollagenous component of cartilage extracellular matrix, but it has also been localized in osteoblasts. COMP counteracts the activity of “extracellular matrix protein 1”, which inhibits the matrix mineralization and endochondral bone formation in the growth plates [54]. The expression of collagen and noncollagenous proteins was decreased in MSCs cultured into the scaffolds. The negative effect was counteracted by the presence of BMP2, which limited the downregulation observed with nanocomposites, and increased remarkably the expression of BGLAP/osteocalcin and POSTN/periostin, while CLEC3B/tetranectin was significantly decreased. Here, we have no sufficient information to explain why CLEC3B behaved differently in comparison to the other noncollagenous proteins. Other Authors found that dexamethasone and TGFβ1 used simultaneously may inhibit the expression of tetranectin in SV-HFO osteoblastic cells [55].

The TNFRS11 expression did not differ between MSCs cultured on the scaffolds and MSCs on TCPS (Figure 3n), but a decrease was observed after the addition of the mineralizing medium in cells exposed to BMP2. The physiological role of osteoprotegerin, which increases during osteoblast differentiation, is the inhibition of osteoclastogenesis, but its expression must decrease in mineralizing osteoblasts when starts the “reverse phase” leading to bone resorption [56]. Therefore, this finding further confirms that the mineralization process is favored by BMP2 released from bioactive nanocomposites.

Wnt signaling related genes. WNT signals are transduced through transmembrane-type WNT receptors encoded by Frizzled (FZD) genes. FZD8 is a frizzled receptor for the canonical Wnt-signaling pathway, which plays a critical role in skeletal development and osteogenesis, promoting the differentiation of mesenchymal cells into osteoblasts, and inhibiting their differentiation into chondrocytes and adipocytes [57]. FZD8 is constantly upregulated during the formation of mineral nodules, even though its function in driving the mineralization process has not been elucidated [8,43]. We confirmed the up-regulation of this receptor after addition of mineralizing agents (Figure 3o); however, no changes were observed whether MSCs were cultured onto TCPS or scaffolds, suggesting that the microenvironment is not relevant in influencing the expression of this receptor.

Even though our data show that gene expression may be influenced by the various nanocomposites, significance following statistical analysis was found only when very large differences were observed. In this regard, a scoring system was conceived for grading the performance of nanocomposites in comparison to TCPS, assuming TCPS as the standard culture condition where the ability of MSC to generate new bone is proven by the deposition of mineral nodules. The score was calculated for each experiment and for each ‘gene of interest/GAPDH’ ratio, and a value was assigned to each comparison according to the following criteria:

**3** = scaffold - to - TCPS equal or higher than 1.1;
**2** = scaffold - to - TCPS from 0.9 to 1.1;
**1** = scaffold - to - TCPS from 0.1 to 0.9;
**0** = scaffold - to - TCPS from −0.1 to 0.1;
−**1** = scaffold - to - TCPS from −0.9 to −0.1;
−**2** = scaffold - to - TCPS from −1.1 to −0.9
−**3** = scaffold - to - TCPS equal or lower than −1.1.

The mean of score values obtained in each experiment for each gene, the sum of the mean scores obtained in both differentiation and mineralization phases, and the total sum of the mean scores are shown in Table 2. Data are shown as increments corresponding to 0.33, representing the accuracy of the measurements, since the mean score of each gene was calculated on the basis of three experiments. The best total score was observed for PLLA + CNT + HA + BMP2, followed by PLLA + CNT + HA and PLLA + CNT + HA + PT with similar values, and finally by PLLA + CNT. No remarkable differences in gene expression were observed during the first 14 days of culture, although the “differentiation score” of PLLA + CNT + HA and PLLA + CNT + HA + BMP2 showed a positive trend in comparison to TCPS. The “mineralization score” was significantly higher in PLLA + CNT + HA + BMP2 than in PLLA + CNT and PLLA + CNT + HA (Wilcoxon test, *p* = 0.005 and *p* = 0.017, respectively), while the difference against PLLA + CNT + HA + PT was not significant (*p* = 0.17).

In our study, PLLA-based scaffolds were found more osteoconductive for human MSCs when micro-hydroxyapatite and, even more, BMP2 were included in the 3D matrix. These findings are in contrast with data from a recent study on similar disc-shaped composites seeded with rat marrow stromal cells [58], where PLLA + CNT + HA was compared with PLLA in presence or not of BMP2. In this study nor micro-hydroxyapatite particles nor BMP2 were found to consistently improve proliferation and osteogenic differentiation of rat precursors. Aside from scaffold shape, which itself may affect the final results, the discrepancy of our data with such results may be ascribed to a number of factors. First, an osteogenic medium with β-glycerophosphate was adopted from the seeding time of cells on the discs, while in our protocol β-glycerophosphate was added after the phase of MSC proliferation, *i.e.*, on cells at 80% confluency. In our opinion the osteogenic induction of MSCs from an early stage of culture may in some way hamper cell proliferation in favor of differentiation, changing the well-known *in vitro* progression of MSCs through proliferation, matrix deposition and mineralization [59]. Second, different cell types may respond differently to the same substrate, and rat MSCs have been shown to be more sensitive to metabolites produced in culture than human MSCs [60]. Concerning the response to BMP2, the induction of different BMP-receptors and the possibility of a different intracellular pathway for rat MSCs compared to human MSCs may account for the dissimilar results [61,62].

## 3. Experimental Section

### 3.1. Scaffold Preparation

PLLA, with an inherent viscosity of 0.90–1.2 dL/g, an average molecular weight (Mw) and polydispersity (Mw/Mn) of 104,000 and 1.80 respectively (measured by gel-permeation chromatography), was supplied by Absorbable Polymers (Pelham, AL) and used as received. The CNTs (90% purity) were supplied by Thomas Swan & Co. Ltd., with diameters of about 2 nanometers and lengths of several microns. Commercial HA powder, with a mean particle size of 5 microns (HA), was purchased from Plasma Biotal (Plasma Biotal, Tideswell, Derbyshire, UK). 1,4-dioxane was obtained from Sigma Aldrich and used as received without further purification.

Porous scaffolds were fabricated using a thermally induced liquid-liquid phase separation technique [63]. PLLA was dissolved in a 87/13 (v/v) mixture of 1,4-dioxane and distilled deionized water to a final concentration of 8% (w/v). To prepare the nanocomposite scaffolds, 0.08% w/v CNT and 0.08% w/v HA were added to 8% w/v PLLA solution, and the mixtures sonicated and warmed at ca. 63 °C in order to disperse the CNT and HA completely. Then, the homogeneous dispersion was fast-frozen at −16 °C overnight to induce liquid-liquid phase separation. Finally, solvents were removed by freeze-drying for six days.

The composites were prepared as three-dimensional scaffolds (3 × 3 × 3 mm) made of 8% w/v PLLA porous matrix with 1% wt CNT added or not with 1% wt HA. Some scaffolds were further added with 100 ng/scaffold BMP2, while other scaffolds were treated with PECVD technique as detailed elsewhere (PT) [64].

### 3.2. Cell Culture

Bone marrow samples were collected by reaming the metaphysis and proximal diaphysis of the femur of three donors (#3002, male, 49 years; #3080, male, 46 years; #3369, female, 75 years), who had been scheduled for reconstructive joint surgery. The sample collection was approved by the Institutional Ethical Committee for human research. MSC were obtained as previously reported [4,43]. Briefly, marrow was filtered to remove bony fragments, diluted, and stratified on Ficoll-Hypaque gradient (Sigma, Milan, Italy) to collect mononuclear cells, which were seeded at a density of 250,000 cell/cm^2^ in α-MEM supplemented with 10% FBS, 100 U/mL penicillin, 0.1 mg/mL streptomycin, and 100 μmol/L ascorbic acid-2 phosphate (Sigma) (control medium). The cultures were incubated at 37 °C in 95% air/5% CO_2_, and after 4 days the adherent cells (MSCs) were detached (T0) and about 15,000 cells (14,727 ± 214) were seeded on the 3 mm^3^ scaffolds with control medium added with 10^−8^ M dexamethasone (differentiation medium). The constructs were maintained in a 96-well plate for 14 days. Flow cytometry analysis confirmed that cells at the first confluence on tissue culture polystyrene (TCPS) expressed typical MSC surface antigens, including high levels of CD44, CD90, CD105, CD166 (>90%), while the percentages of CD45 and CD117 were very low (<1%) (Instrumentation Laboratory, Milan, Italy) [65].

In order to analyze the mineralization ability of MSCs, about 100,000 cells (95,333 ± 2,353) from the second confluence were seeded on the PLLA scaffolds or on TCPS, and cultured for 7 days in medium containing 10 mM β-glycerophosphate (Sigma) (mineralization medium). At the final time point, the formation of mineral nodules in cultures on TCPS was assessed. Morphological, biochemical and molecular assays were performed before the seeding of MSCs on nanocomposites (T0), in differentiation medium (7 days-TD1 and 14 days-TD2 from the seeding), and in mineralization medium (before cell seeding onto nanocomposites-TM0 and after 7 days-TM1).

### 3.3. Morphological Assays

Density and morphology of MSCs on TCPS was monitored by light microscopy throughout the culture period, until the mineral deposition (data not shown).

Morphology and spreading of cells on the scaffolds was observed by scanning electron microscopy (SEM). The samples were fixed with Karnovsky’s fixative (2% paraformaldehyde, 2.5% glutaraldehyde and 0.1 M sodium cacodylate buffer, pH 7.2–7.4) for 1 h, then post-fixed in 1% osmium tetroxide, dehydrated in ascending ethanol concentrations and dried in hexametildisilazane. All specimens were then coated with palladium-gold before being examined in a Philips SEM 515 at a voltage of 15 kV.

### 3.4. Biochemical Assays

Biochemical analyses were performed at TD1, TD2, and TM1. The number of cells within the scaffolds was determined using the Picogreen assay (Quant-IT Picogreen dsDNA, Life technologies, Monza, Italy). Cells were lysed with 0.01% sodium dodecyl sulphate and sonication, and 10 μL of cell lysate or standard were mixed with 10 μL of Picogreen solution in wells of a 96-well plate. The fluorescence was read at 480–520 nm with a microplate fluorescence reader CytoFluor 2350 (Millipore Corp., Bedford, MA, USA). The mean DNA content of the cells was defined by interpolation of values from a standard curve, then the number of cells was calculated from the Picogreen test results.

The number of cells recovered from cultures at each endpoint was calculated by interpolating the DNA content with a standard curve, and extrapolating how many MSCs colonized the scaffold. Data were “ln” transformed to better highlight the average deviations from the baseline (= 0), represented by the number of seeded cells.

Cell viability was assessed by the Alamar Blue test (Serotec Ltd, Oxford, UK), by following the manufacturer’s recommendations. Briefly, the Alamar Blue solution was added (10% v/v) to the culture. After incubation for 4 h at 37 °C the fluorescence was measured using the microplate fluorescence reader, with an excitation wavelength of 490 and an emission wavelength of 530 nm. The results were expressed as relative fluorescence units (RFUs).

Alkaline phosphatase (ALP) was measured using a biochemical method (Sigma, N7653) in cell lysates obtained with 0.01% SDS, and the ALP activity expressed as nanomoles of *p*-nitrophenol formed per minute normalized to the cell number, as assessed from DNA content.

The synthesis of type I collagen was assessed by measuring its metabolic product released in the culture supernatant. Levels of C-terminal propeptide of type I collagen (CICP) were quantified by enzyme immunoassay, according to the manufacturer’s instructions (Quidel Corporation, Heidelberg, Germany).

### 3.5. Gene Expression Analysis

The gene expression of MSCs was evaluated at TM0, *i.e.*, before cell seeding onto nanocomposites for mineralization, and at TD1, TD2, and TM1 after cell seeding. At fixed time points, cells were collected and total RNA isolated on RNeasy micro and mini kits (Qiagen, GmbH, Hilden, Germany), following the manufacturer’s recommendations. RNA was treated with DNase (Qiagen), eluted in 14 μL of RNase-free water and stored at −80 °C. RNA concentration and purity were determined by spectrophotometry (NanoDrop^®^ ND-1000, NanoDrop technologies). The retrotranscription was performed with MuLV Reverse Transcriptase (Applied Biosystems, Foster City, CA, USA), according to the manufacturer’s instructions. Real time PCR was performed by using the Light Cycler instrument and the Universal Probe Library system (Roche Applied Science, Monza, Italy) [66]. ProbeLibrary probes and primers were selected using a web-based assay design software (Probe Finder) [67]. 1 μg of cDNA was amplified, and the corresponding threshold cycle was referred to an eight-point standard curve. An housekeeping gene (GAPDH) was used as a references to normalize Real Time PCR data, which were expressed as ratio of “gene of interest” to GAPDH.

### 3.6. Statistical Analysis

Statistical analysis was performed using StatView 5.01 for Windows software (SAS Institute Inc., Cary, NC). Data were expressed as arithmetic mean plus and minus the standard error of the mean (SEM) of three separate experiments. A paired analysis of the data (Wilcoxon signed rank test) was applied to detect the effects of the treatments. Because of the low number of experiments, the results analysed with the “Wilcoxon Rank Sum test” may produce misleading results. In particular, we can fail in rejecting the null hypothesis when the null hypothesis is true, *i.e.*, the addition of biomimetic signals to the nanocomposite has no consequences. In order to minimize the type I error rate, the significance level was fixed at 0.05.

In order to quantify objectively the gene expression results, a scoring system was conceived for grading the performance of nanocomposites in comparison to TCPS. The calculation was performed using a “Microsoft^®^ Excel 2002” file, and a pivot table (Table 2) was obtained to summarize the mean of score values obtained in each experiment for each gene, the sum of the mean scores obtained in both differentiation and mineralization phases, and the total sum of the mean scores.

## 4. Conclusions

In this study the osteoconductive properties of four three-dimensional PLLA-based composites were evaluated by using a combination of morphological, biochemical and molecular assays. Collectively, our results show that human MSC adhesion, proliferation and osteogenic differentiation are modulated by the extracellular cues introduced in the polymeric 3D scaffolds. Particularly, CNT-added PLLA scaffold has a low osteoconductive ability, because neither adhesion nor proliferation or bone cell differentiation were promoted. Therefore, carbon nanotubes, though efficient in strengthening the polymeric matrix, do not provide MSCs with specific signals. In contrast, the addition of HA particles improves the performance of the PLLA + CNT composite, and plasma treatment further enhances MSC adhesion to the surface, as well as their proliferation and differentiation. The best results in terms of bone formation *in vitro* were obtained with the loading of BMP2 on the composite, as mineralization was promoted more than with any other scaffold. It may be suggested that the already osteoconductive PLLA/CNT/HA scaffold becomes “more osteoinductive”, too, when BMP2 is included. Indeed, the enrichment of the polymeric matrix with biomimetic signals enables a closer matching of scaffolds to the *in vivo* environment, and their sinergy strongly enhances the response of adult mesenchymal cells.

## Figures and Tables

**Table 1:** List of selected genes.

Gene Symbol	Gene	Function	Expression in TCPS cultures [43]
ALPL	Alkaline phosphatase liver/bone/kidney	Membrane bound glycosylated enzyme involved in matrix mineralization.	↑ TD2
BGLAP	Bone gamma-carboxyglutamate protein (Osteocalcin)	Noncollagenous matrix protein is associated the calcium phosphate mineral phase of bone. BGLAP is the only gene that is expressed in osteoblasts but not in other cells.	↑ TD2
CLEC3B	Tetranectin	Matrix protein (plasminogen-binding) involved in mineralization process.	↑ TD2
COL12A1	Type 12 collagen, alpha 1 chain	Type 12 collagen is found in association with type 1 collagen, an association that is thought to modify the interactions between collagen 1 and the surrounding matrix.	↑ TD1, ↑ TD2
COL1A1	Type 1 collagen, alpha 1 chain	Type 1 collagen is a fibril-forming collagen found in most connective tissues and is abundant in bone, cornea, dermis and tendon. It comprises two α1 chains and one α2 chain.	↑ TD1, ↑ TD2
COMP	Cartilage oligomeric matrix protein	Noncollagenous ECM protein; it is expressed in the hypertrophic chondrocytes and in osteoblasts around developing bone.	↑ TD1, ↑ TD2, ↑ TM1
FZD8	Frizzled homolog 8	Receptor for the Wingless type MMTV integration site family of signaling proteins.	↑ TM1
IBSP	Bone sialoprotein	Noncollagenous glycoprotein expressed in mineralized tissues; it mediates cell-to-matrix attachment and binds to calcium and HA.	↑ TD1, ↑ TD2, ↑ TM1
POSTN	Periostin	Secreted protein expressed during osteoblastic differentiation and maturation and abundantly found in mineralized bone nodules *in vitro*.	↑ TD1, ↑ TD2
Runx2	Runt-related transcription factor 2	Trascription factor belonging to the TGFβ signaling pathway; it is considered a master regulatory switch to address the commitment of MSC to osteoblastic differentiation and skeletal morphogenesis.	↑ TD1, ↑ TD2
Smad4	Mothers against decapentaplegic homolog 4	Smad 4 is a common partner of BMP- and TGFβ-receptor Smads; Smad4 induces expression of Runx2 and Osterix in osteoprogenitor cells.	↑ TD1
SP7	Sp7 transcription factor (Osterix)	SP7 is a transcription factor which acts downstream of Runx2 to induce osteoblastic differentiation in osteochondroprogenitor cells. Sp7 is responsible for the activation of BGLAP and COLA1 genes.	↑ TD1
SPARC	Osteonectin	Matrix-associated protein expressed in bone remodeling areas; it regulates angiogenesis and cell-matrix interactions.	↑ TD1, ↑ TD2
THBS1	Thrombospondin 1	THBS1 is a negative regulator of TGFβ signaling. It co-localizes with TGFβ and mediates cell-to-cell and cell-to-matrix interactions.	↑ TD1, ↑ TD2
TNFRSF11B	Tumor necrosis factor receptor superfamily, member 11b (osteoprotegerin)	Osteoblast-secreted decoy receptor that functions as a negative regulator of bone resorption.	↑ TD2

**Table 2:** Mean of score values (*n* = 3) calculated for each gene.

Process	Gene	PLLA+CNT	PLLA+CNT+HA	PLLA+CNT+HA+BMP	PLLA+CNT+HA+PT
**Differentiation**					
	ALP	−2.00	0.00	−1.33	−1.33
	BGLAP	0.67	0.33	1.33	1.00
	CLEC3B	0.33	−0.33	−0.33	−0.33
	COL12A1	−0.33	−0.33	0.33	−0.67
	COL1A1	0.33	0.33	−0.33	0.33
	COMP	1.33	0.33	0.00	−0.33
	IBSP	−0.33	−0.33	0.00	−0.67
	Osx	−2.00	−0.67	−0.33	−2.00
	POSTN	0.33	0.33	0.33	0.00
	RUNX2	−2.67	0.33	0.67	−0.67
	Smad4	0.00	0.33	0.33	1.00
	SPARC	0.00	−0.33	−0.33	−0.33
	THS1	0.33	0.33	0.33	0.33
	TNFRS11	0.33	0.33	−0.33	1.00
**Differentiation Score**^a^		**−3.67**	**0.67**	**0.33**	**−2.67**

**Mineralization**					
	ALP	−1.67	−0.67	−1.67	−1.00
	BGLAP	−0.33	−0.33	0.67	−0.33
	CLEC3B	−1.00	−1.33	−1.67	−0.67
	COL12A1	−1.00	−1.00	−0.33	−1.00
	COL1A1	−1.67	−1.33	−1.00	−1.33
	COMP	−2.00	−1.67	−0.33	−2.00
	FZD8	0.33	−0.67	−0.33	−0.33
	IBSP	−2.00	−2.00	−0.33	−1.67
	POSTN	−0.33	−0.67	1.00	−1.00
	SPARC	−1.33	−1.00	−0.33	−1.00
	TNFRS11	−0.33	−1.00	−1.00	1.00
**Mineralization Score**^b^		**−11.33**	**−11.67**	**−5.33**	**−9.33**
**Total score**^c^		**−15.00**	**−11.00**	**−5.00**	**−12.00**

a = sum of the mean scores in differentiation;

b = sum of the mean scores in mineralization;

c = total sum of the mean scores.
